# Supplementary material for: Enhancing Mild Cognitive Impairment Auxiliary Identification Through Multimodal Cognitive Assessment with Eye Tracking and Convolutional Neural Network Analysis
Source: Biomedicines. 2025 Mar 18;13(3):738. doi: 10.3390/biomedicines13030738 (PMC11940729; doi:10.3390/biomedicines13030738)
Supplement: Supplementary file 1 [file biomedicines-13-00738-s001.zip › biomedicines-3466565-supplementary.pdf]

Supplementary Analyses

Table S1 Definitions of Input Features

Task Parameter	Explanation
	Pro- and anti-saccades task
Accuracy	the number of trials with initial eye movement response to the target divided by the total remaining number of trials
Latency	the time from target onset to saccade initiation
Gain	the deviation when participant look at the target stimulus, and was calculated as a percent error: 100%×absolute value of (1- [amplitude of the initial saccade /target amplitude])
Fixation dispersion	the variance when participant focused on the fixation point
	Smooth pursuit task
Delay	the difference between the trigonometric function curve which fitted by eye movement trajectory and the target curve on the x-axis
Saccade compensation	the number of saccades during smooth pursuit
Fixation dispersion	the variance when participant focused on the fixation point
	Memory-guided task
Gain	the deviation when participant look at the target stimulus, and was calculated as a percent error: 100%×absolute value of (1- [amplitude of the initial saccade /target amplitude])
Fixation dispersion	the variance when participant focused on the fixation point
	Predictive saccade task
Latency	the time from target onset to saccade initiation
Gain	the deviation when participant look at the target stimulus, and was calculated as a percent error: 100%×absolute value of (1- [amplitude of the initial saccade /target amplitude])
Fixation dispersion	the variance when participant focused on the fixation point

Table S2 Comparison of elderly and young adults

Task parameter	Elderly groups		Young	*t value*（95）	*t value*（126）
	Control	MCI	group	*(Control-MCI)*	*（Elderly-Young）*
			The pro- and anti- saccades tasks		
Total accuracy (%)	0.42	0.39	0.83	1.54	-22.03***
Pro-saccade accuracy (%)	0.45	0.42	0.89	1.49	-23.24***
Anti-saccade accuracy (%)	0.38	0.36	0.78	0.92	-15.15***
Mean latency (ms)	0.30	0.25	0.22	1.51	2.95**
Pro-saccade latency (ms)	0.29	0.24	0.22	1.43	2.86**
Anti-saccade latency (ms)	0.30	0.25	0.23	1.08	2.06*
Mean gain (%)	1.01	0.82	0.53	0.69	1.69
Pro-saccade gain (%)	1.06	0.85	0.44	0.59	1.78
Anti-saccade gain (%)	0.96	0.79	0.62	0.83	1.48
Fixation dispersion (%)	0.03	0.02	0.01	0.89	3.83***
			Smooth pursuit task		
Mean variance	0.63	0.58	0.76	1.12	-4.82***
Variance vertical at 0.25Hz	0.68	0.64	0.86	0.68	-5.97***
Variance vertical at 0.4Hz	0.65	0.59	0.81	1.34	-5.77***
Variance horizontal at 0.25Hz	0.60	0.59	0.71	0.20	-3.30**
Variance horizontal at 0.4Hz	0.59	0.51	0.63	1.60	-1.43
Mean delay	0.23	0.26	0.18	-0.68	2.73**
Delay vertical at 0.25Hz	0.16	0.17	0.09	-0.35	3.33**
Delay vertical at 0.4Hz	0.27	0.30	0.23	-0.69	1.66
Delay horizontal at 0.25Hz	0.19	0.18	0.10	0.10	3.32**
Delay horizontal at 0.4Hz	0.30	0.36	0.28	-0.84	0.64
Saccade compensation	23.97	24.13	26.33	-0.27	-5.57***
Fixation dispersion	0.04	0.03	0.02	1.41	1.82
			Memory-guided task		
Mean gain (%)	0.75	0.74	0.25	0.10	11.38***
Gain at 5°(%)	0.86	0.86	0.32	-0.01	7.98***

Gain at 10°(%)	0.73	0.71	0.23	0.24	10.16***
Gain at 15°(%)	0.64	0.64	0.20	0.05	9.95***
Fixation dispersion	0.04	0.04	0.04	0.73	-0.13
Predictive saccade task					
Mean variance	0.40	0.37	0.59	1.15	-6.94***
Mean latency (ms)	0.25	0.26	0.19	-0.38	5.29***
Mean gain (%)	0.87	0.60	0.49	0.87	1.01
Fixation dispersion	3.24	0.08	0.07	0.83	0.57
Behavioral task					
Forward visuospatial task	3.43	3.17	5.17	1.74	-11.66***
Backward visuospatial task	3.29	3.10	5.09	1.59	-12.28***
Forward digit span task	5.48	5.03	9.32	1.97	-17.03***
Backward digit span task	3.86	3.23	8.01	3.42**	-13.62***
Stroop consistent accuracy	0.98	0.99	0.99	-1.28	-0.80
Stroop inconsistent accuracy	0.95	0.96	0.97	-0.56	-1.15
Stroop consistent response time	1237.97	1169.26	622.15	0.93	12.54***
Stroop inconsistent response time	1397.66	1387.38	695.67	0.11	13.10***

Note: *p < 0.05; **p < 0.01; ***p < 0.001.

Table S3 Correlation between eye movement variables and behavioral features

	Forward visuospatial task	Backward visuospatial task	Forward digit span task	Backward digit span task	Stroop consistent accuracy	Stroop inconsistent accuracy	Stroop consistent response time	Stroop inconsistent response time
The pro- and anti- saccades tasks								
Total accuracy (%)	.424**	.532**	.549**	.507**	-.045	-.031	-.360**	-.391**
Pro-saccade accuracy (%)	.450**	.502**	.512**	.545**	-.008	-.016	-.393**	-.436**
Anti-saccade accuracy (%)	.412**	.546**	.555**	.466**	-.063	.019	-.378**	-.387**
Mean latency (ms)	-.085	-.127	-.125	-.049	.045	.044	-.005	.020
Pro-saccade latency (ms)	-.071	-.142	-.126	-.058	-.031	-.025	.062	.082
Anti-saccade latency (ms)	-.010	.003	-.039	.037	.093	.130	-.095	-.056
Mean gain (%)	-.375**	-.363**	-.297**	-.316**	-.053	-.090	.377**	.374**
Pro-saccade gain (%)	-.471**	-.464**	-.385**	-.451**	-.106	-.078	.442**	.452**

Anti-saccade gain (%)	-.259**	-.237**	-.173	-.153	.031	-.124	.281**	.263**
Fixation dispersion (%)	-.354**	-.385**	-.256**	-.265**	-.062	-.153	.305**	.382**
			Smooth pursuit task					
Mean variance	.335**	.321**	.146	.268**	-.081	.050	-.266**	-.264**
Variance vertical at 0.25Hz	.406**	.399**	.250**	.369**	-.141	-.012	-.289**	-.315**
Variance vertical at 0.4Hz	.307**	.360**	.254**	.340**	-.066	-.072	-.287**	-.287**
Variance horizontal at 0.25Hz	.259**	.210*	.074	.175*	-.118	.042	-.220*	-.212*
Variance horizontal at 0.4Hz	.225*	.197*	.014	.090	-.021	.125	-.168	-.146
Mean delay	-.250**	-.150	-.006	-.058	.024	-.106	.190*	.174*
Delay vertical at 0.25Hz	-.241**	-.159	-.085	-.180*	.079	-.004	.265**	.250**
Delay vertical at 0.4Hz	-.183*	-.110	-.079	-.081	-.003	-.147	.157	.145
Delay horizontal at 0.25Hz	-.163	-.115	.006	-.106	-.043	-.119	.156	.145
Delay horizontal at 0.4Hz	-.131	-.117	.077	.073	.010	-.027	.027	.023
Saccade compensation	.344**	.328**	.255**	.289**	-.020	.064	-.237**	-.215*
Fixation dispersion	-.267**	-.245**	-.011	-.133	.103	.033	.168	.130
			Memory-guided task					
Mean gain (%)	-.521**	-.562**	-.494**	-.508**	.047	-.063	.550**	.534**
Gain at 5° (%)	-.453**	-.486**	-.428**	-.426**	.098	-.023	.456**	.474**
Gain at 10° (%)	-.570**	-.594**	-.526**	-.564**	.000	-.063	.584**	.556**
Gain at 15° (%)	-.540**	-.570**	-.508**	-.549**	.013	-.135	.549**	.512**
Fixation dispersion	-.096	-.055	.088	.074	.045	-.089	.036	.042
			Predictive saccade task					
Mean variance	.425**	.467**	.377**	.460**	.056	.045	-.503**	-.474**
Mean latency (ms)	-.277**	-.298**	-.299**	-.370**	-.129	-.105	.441**	.454**
Mean gain (%)	-.192*	-.221*	-.180*	-.184*	-.096	.040	.181*	.163
Fixation dispersion	-.152	-.153	.033	-.055	-.014	.120	.042	.044

Note: *p < 0.05; **p < 0.01; ***p < 0.001.

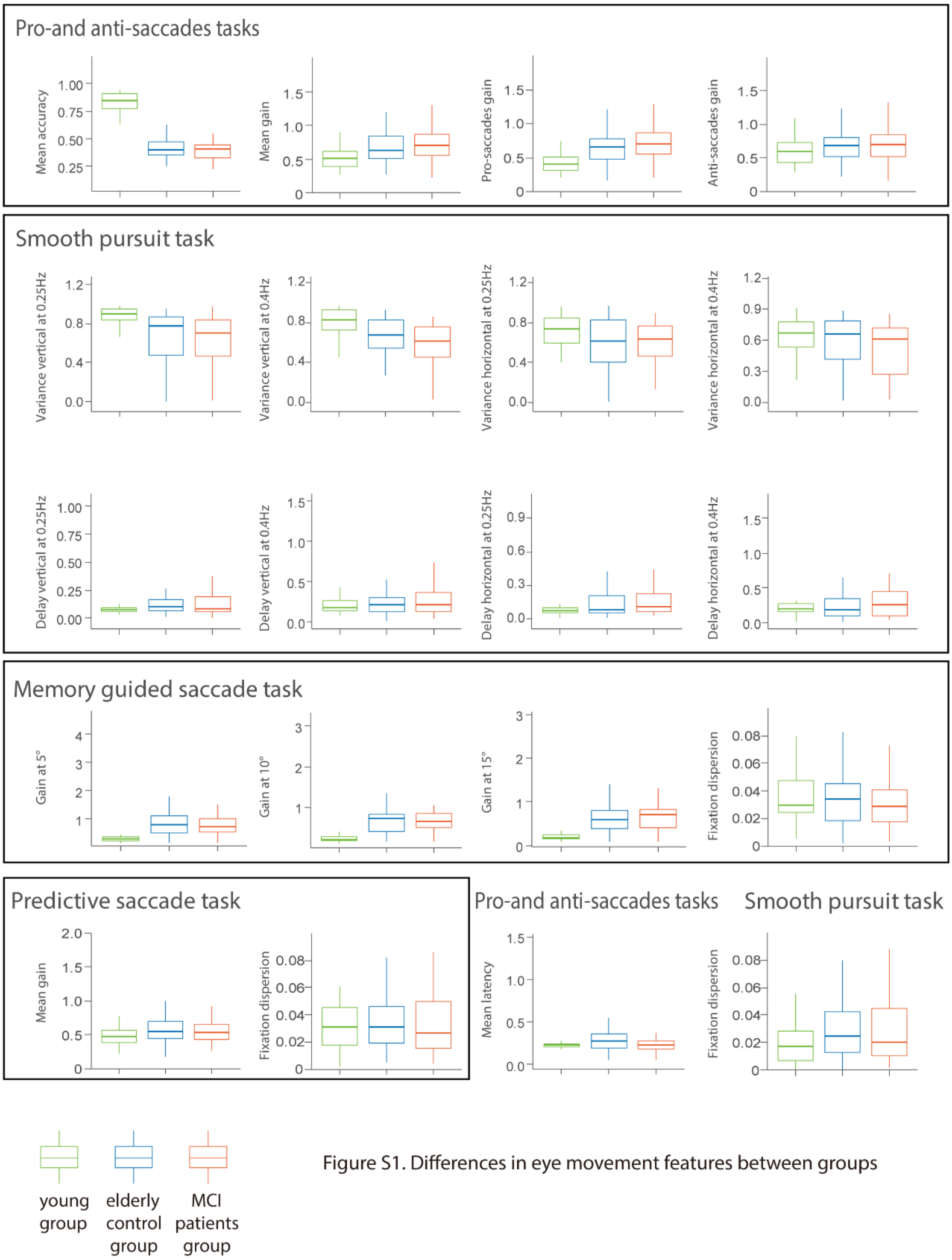

Figure S1. Differences in eye movement features between groups

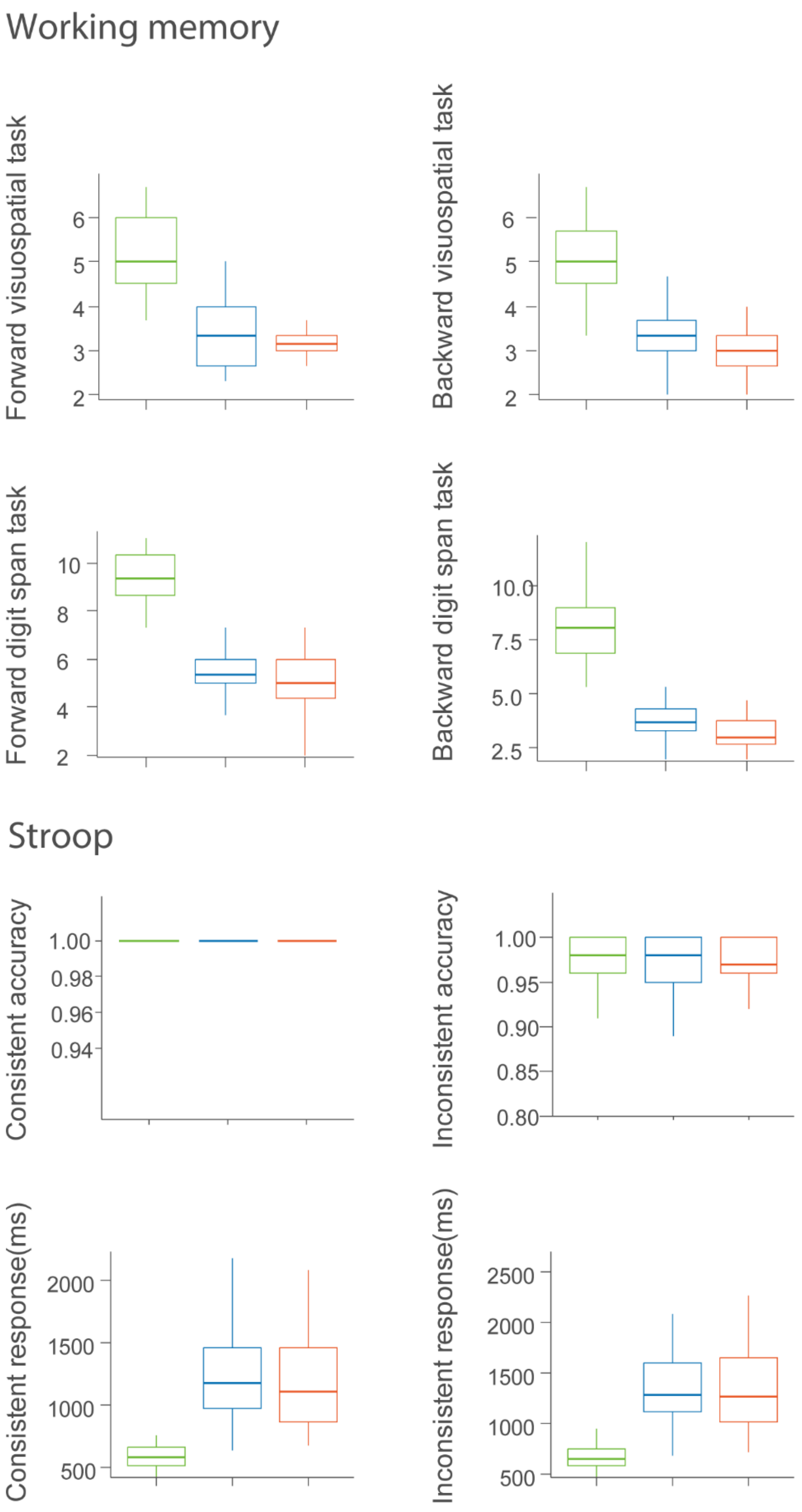

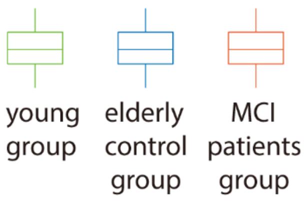

Figure S2. Differences in behavioral features between groups
